# Potential Role of CXCL10 in Monitoring Response to Treatment in Leprosy Patients

**DOI:** 10.3389/fimmu.2021.662307

**Published:** 2021-07-20

**Authors:** Helen Ferreira, Mayara Abud Mendes, Mayara Garcia de Mattos Barbosa, Eliane Barbosa de Oliveira, Anna Maria Sales, Milton Ozório Moraes, Euzenir Nunes Sarno, Roberta Olmo Pinheiro

**Affiliations:** ^1^ Leprosy Laboratory, Oswaldo Cruz Institute, Oswaldo Cruz Foundation, Rio de Janeiro, Brazil; ^2^ Cascalho-Platt Laboratory, Department of Surgery, University of Michigan, Ann Arbor, MI, United States

**Keywords:** leprosy, skin cells, IFN-γ, CXCL-10, multidrug therapy

## Abstract

The treatment of multibacillary cases of leprosy with multidrug therapy (MDT) comprises 12 doses of a combination of rifampicin, dapsone and clofazimine. Previous studies have described the immunological phenotypic pattern in skin lesions in multibacillary patients. Here, we evaluated the effect of MDT on skin cell phenotype and on the *Mycobacterium leprae*-specific immune response. An analysis of skin cell phenotype demonstrated a significant decrease in MRS1 (SR-A), CXCL10 (IP-10) and IFNG (IFN-γ) gene and protein expression after MDT release. Patients were randomized according to whether they experienced a reduction in bacillary load after MDT. A reduction in CXCL10 (IP-10) in sera was associated with the absence of a reduction in the bacillary load at release. Although IFN-γ production in response to *M. leprae* was not affected by MDT, CXCL10 (IP-10) levels in response to *M. leprae* increased in cells from patients who experienced a reduction in bacillary load after treatment. Together, our results suggest that CXCL10 (IP-10) may be a good marker for monitoring treatment efficacy in multibacillary patients.

## Introduction

Multibacillary leprosy (MB) patients are responsible for the active transmission of the disease in the community (1). Previous studies have demonstrated that macrophages present in skin lesions of MB patients have the characteristics of anti-inflammatory macrophages, with higher phagocytic properties and increased bacterial survival. These are furthermore mediated by an increased expression of proteins involved in host iron metabolism and they contribute to a higher bacillary load in these patients (2–4).

MB patients do not mount an effective cellular-mediated response to *Mycobacterium leprae* and may present numerous and symmetrically distributed lesions, with an increased ratio of CD8:CD4 T cells and increased production of Th2 cytokines such as IL-4, IL-5 and IL-10 (5). These Th2 cytokines are associated with the production of antibodies that do not confer protection against the disease. IL-10 is associated with increased phagocytic activity in skin macrophages from MB patients, as well as with a reduction in the antimicrobial properties of host cells, which are responsible for the maintenance of *M. leprae*-susceptible macrophages (6–8).

Until now, a specific drug for the treatment of leprosy was not available (9). Leprosy is a bacterial disease caused by *M. leprae*, an intracellular pathogen that infects keratinocytes, macrophages and histiocytes in the skin and Schwann cells in the peripheral nerves (10–12). MB treatment consists of a combination of rifampicin, clofazimine and dapsone in 12 doses (13). Although patients are considered “cured” after the completion of MDT, leprosy reactions, permanent disability and occasional relapse/reinfection have been observed in many patients (14). Moreover, not all patients present a reduction in bacillary load after the 12 doses of MDT.

Several studies evaluated the impact of MDT on immune response. MDT changes the profile of serum cytokines in *M. leprae*-infected patients (15, 16), and a reduction in antibody response in MB patients was considered a parameter for monitoring MDT effectiveness (17). The immunosuppressive and anti-inflammatory properties of MDT are associated with the inhibition of some cell types, and they do not seem to interfere directly in the production of mediators and cytokines (18–21). In addition, during MB clinical courses, there are disturbances in skin lipid metabolism that are modulated by MDT (22).

Here, we investigated the expression of cell markers associated with an anti-inflammatory phenotype in macrophages in skin lesion cells from MB patients that presented or showed no reduction in bacillary load after MDT. Furthermore, we evaluated whether MDT was able to restore the cellular immune response against *M. leprae* in these patients.

## Materials and Methods

### Study Population

In this study, we utilized biological samples from a total of 55 adult patients. The patients were men and women between 18 and 81 years old who had been diagnosed with MB leprosy and who were categorized according to Ridley and Jopling’s classification (23) as being lepromatous-lepromatous (LL), meaning they had no reaction at the onset or at the completion of treatment (release) (**Table 1**). Patients under the age of 18 with comorbidities such diabetes, hepatitis, syphilis and diseases caused by other mycobacteria, as well as patients co-infected with the human immunodeficiency virus and relapse cases were excluded. All patients enrolled were treated at the Souza Araújo Outpatient Unit at FIOCRUZ, Rio de Janeiro, Brazil. Among the recruited patients, 48 received the standard regimen of multidrug therapy (WHO-MDT): rifampicin, dapsone and clofazimine, and 7 received an alternative scheme: rifampicin, clofazimine and ofloxacin. Both groups took these regimens for twelve months. Whole blood and skin lesion samples were obtained at diagnosis, or prior to treatment (onset), and at the conclusion of treatment (release) from patients who did not exhibit any signs of leprosy at both points. Blood samples were used in the study of gene expression and for cytokine measurements, both in whole blood and in serum. Skin lesion samples were obtained with a 6 mm punch and cleaved into two fragments. One fragment was used for histopathological processing and staining by the Hematoxylin-Eosin and Wade methods in order to diagnose cases, while the second fragment was immediately frozen by immersion in liquid nitrogen and used for immunohistochemistry or real-time PCR. Blood without anticoagulants was also collected to obtain serum samples.

**Table 1:** Demographic and clinical data of patients included in the study (n = 55).

Gender (n,%)	
Female	11 (20.0%)
Male	44 (80.0%)
Age (range, min–max)	48 (18 – 81)
BI onset (range, min–max)	4.61 (3.0 – 6.0)
BI release (range, min–max)	3.78 (1.0 – 6.0)
LBI onset (range, min–max)	5.01 (2,3 – 6.0)
LBI release (range, min–max)	2.78 (0,0 – 5,85)
**Reaction during treatment (n,%)**	
Yes	12 (21.82%)
No	43 (78.18%)
**Treatment (n,%)**	
Multidrug therapy (MDT)	48 (87.28%)
Alternative scheme	07 (12.72%)
**BI Reduction (n,%)**	
Yes (WR)	22 (40.0%)
No (NR)	33 (20.0%)

BI, bacillary index; LBI, logarithmic bacillary index of skin lesions; WR, with reduction; NR, no reduction.

### Ethics Statement

This study was carried out in accordance with institutional research ethics committee approval and in Resolution 466/12 of the National Health Council (CAAE 76328517.2.0000.5248, approval number 2.450.910). All volunteers agreed to participate and signed a free and informed consent form prior to their inclusion in the study and any sample collection. All the patients received clinical treatment, follow-up appointments and all information, regardless of their participation or exclusion from the study.

### Immunohistochemistry

Frozen sections of skin lesion samples were examined with a Leica LM1850UV cryostat (Leica, Wetzlar, Germany). The 5 μm thick sections were fixed in cold acetone and hydrated in 0.01M phosphate buffer saline (PBS). Endogenous peroxidase was blocked in 0.3% hydrogen peroxide solution diluted in 0.01M PBS and then washed in 0.01M PBS. Unspecific binding sites were blocked with 0.01M PBS solution containing 10% normal goat serum (NGS) and 0.1% bovine serum albumin (BSA). The following primary antibodies were diluted in 0.01M PBS solution containing 1% NGS and incubated overnight at 4°C in humid chamber: anti-CD68 (1:100 Dako M0814), anti-CD163 (1:25 R&D Systems MAB1607), anti-arginase 1 (BD Transduction labs 610708), anti-IDO (1:100 Millipore MAB1009), anti-MRS-1 (SR-A, Santa Cruz, SC-166184), anti-IFN-γ (1:50 BD Biosciences N554548) and anti-CXCL10/IP-10 (1:50 Santa Cruz SC-101500). Next, sections were washed with 0.01M PBS and incubated in a HiDef signal amplifier solution for 20 min, and then washed in 0.01M PBS and incubated in a HiDef HRP polymer detector solution (kit HiDef detection HRP polymer system, Cell Marque, 954-D) for 20 minutes. Sections were washed twice with 0.01M PBS. Immunostainings were developed in 3-amino-9-ethylcarbazole solution (AEC substrate Kit, Vector Labs SK-4200). The cell nuclei were stained with Harris’ Hematoxylin. Sections were mounted with coverslips using an aqueous mounting medium (Abcam 128982) and the results were analyzed under Nikon Eclipse E400 optical microscope with a plan-apochromatic 20X/0,40 objective (Nikon Instruments Inc., New York, USA).

### Whole Blood Assay

Venous blood samples collected in tubes containing heparin were diluted in serum-free AIM-V medium (GIBCO 12055) in a 1:10 ratio and distributed onto plates in three series of 300 μL in triplicate. A fraction was incubated with sonicated *M. leprae* cell antigens at a 10 μg/mL concentration (BEI Resources, NR19329) and the second aliquot was stimulated with phytohemagglutinin (PHA Sigma 1668) solution at 25 μg/mL as a positive control. Negative controls were non-stimulated cells. Samples were cultured at 37°C in an environment containing 5% CO_2_ for five days. Subsequently, the supernatant was collected and IFN-γ and CXCL10/IP-10 were measured by ELISA (eBiosciences, San Diego, CA, USA).

### ELISA

Serum samples obtained from blood without anticoagulants or supernatants from whole blood cultures were used to determine the concentration of IFN-γ, IL-6, IL-10, IL-17A, TNF, IL-1β, TGF-β and CXCL10/IP-10 cytokines by ELISA, following the manufacturer’s protocol (eBiosciences, San Diego, CA, USA).

### RT-PCR

RNA was extracted from skin lesion fragments by the TRIzol method (Life Technologies15596-018), following the manufacturer’s instructions. To avoid genomic DNA contamination, the RNA was treated with DNAse (RTS DNase Kit, MO BIO Laboratories); integrity was analyzed *via* 1.2% agarose gel electrophoresis. A SuperScript III First-Strand Synthesis System (Life Technologies, 18080-051) was used to perform the reverse transcription. mRNA expression of *CD163, ARGINASE1, MRS1, IDO1, NOS2A, IL15, IFNG* and *CXCL10* was evaluated using TaqMan Fast Universal PCR Master Mix (2X) (Applied Biosystems 4352042) in a StepOnePlus real-time PCR system (Applied Biosystems, MA, USA). All primers were acquired from ThermoFisher Scientific (4331182). The 2^−ΔCT^ method was used to analyze gene expression data using glyceraldehyde-3-phosphate dehydrogenase (*GAPDH*; Hs02758991_g1, Thermo-Fisher Scientific) as a reference gene.

### Statistical Analysis

Statistical significance was calculated by Mann–Whitney or Kruskal–Wallis tests with Dunn’s multiple comparison post-test *via* GraphPad Prism 8.0 software (GraphPad, La Jolla, CA, USA). A *p* ≤ 0.05 was deemed statistically significant.

## Results

### MDT Contributes to Reducing the Anti-Inflammatory Profile of Skin Macrophages in LL Patients

**Figure 1:**
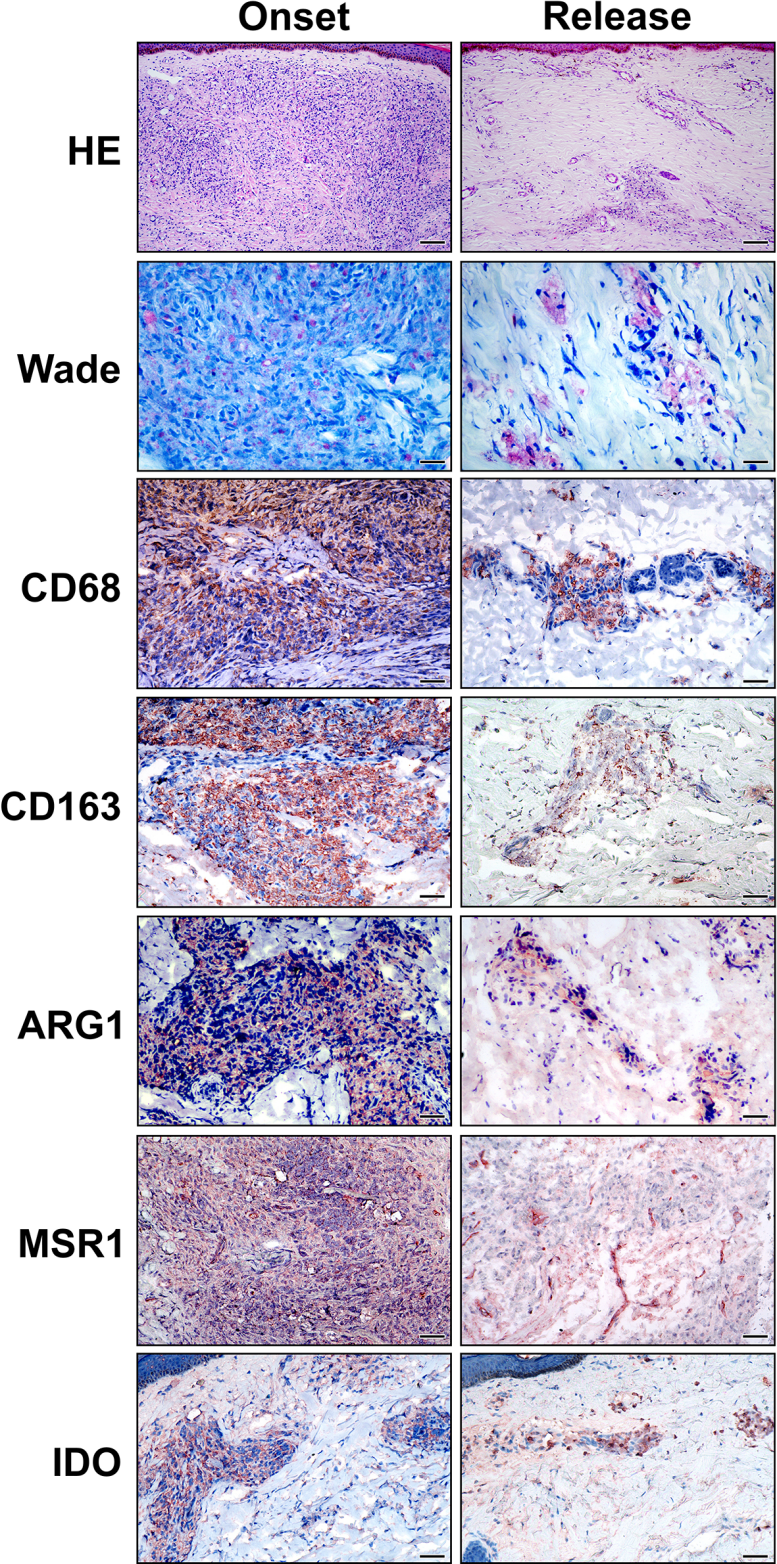
Anti-inflammatory macrophage phenotype in LL skin lesions is reduced at 12-dose MDT release. Skin lesions fragments were collected at LL diagnosis (onset) and at release of 12-dose MDT. Routine Hematoxylin-Eosin (HE) and Wade staining were performed to characterize the inflammatory infiltrate and to verify the presence of alcohol-acid-resistant bacilli in the skin lesions. Additionally, CD68, CD163, Arginase 1 (ARG1), MSR1 (SR-A) and IDO immunostainings were performed to characterize the phenotype of the cells infiltrating the skin lesions. Representative images are shown. Bars: 100 μm (HE), 25 μm (Wade) and 50 µm (immunostaining).

Routine histopathological analyses of skin lesion fragments were performed to select representative specimens of the LL polar form of the disease, both before treatment and after the release of MDT. As previously described (24, 25), the histopathology of lepromatous leprosy is characterized by collections and sheets of macrophages diffusely distributed in the dermis, with few lymphocytes and plasma cells (**Figure 1**). Macrophages present a foamy appearance and are filled with bacilli (**Figure 1**). After 12 doses of MDT, histopathology was variable between the recruited cases, but a reduction in the infiltrate was a common finding in patients who did not develop reactional episodes during treatment (**Figure 1**). A diffuse lymphocytic infiltrate was also observed, and only few foamy cells were observed (**Figure 1**). Immunohistochemistry was performed to identify whether MDT decreases the anti-inflammatory (M2) profile in cells from treated LL patients.

**Figure 2:**
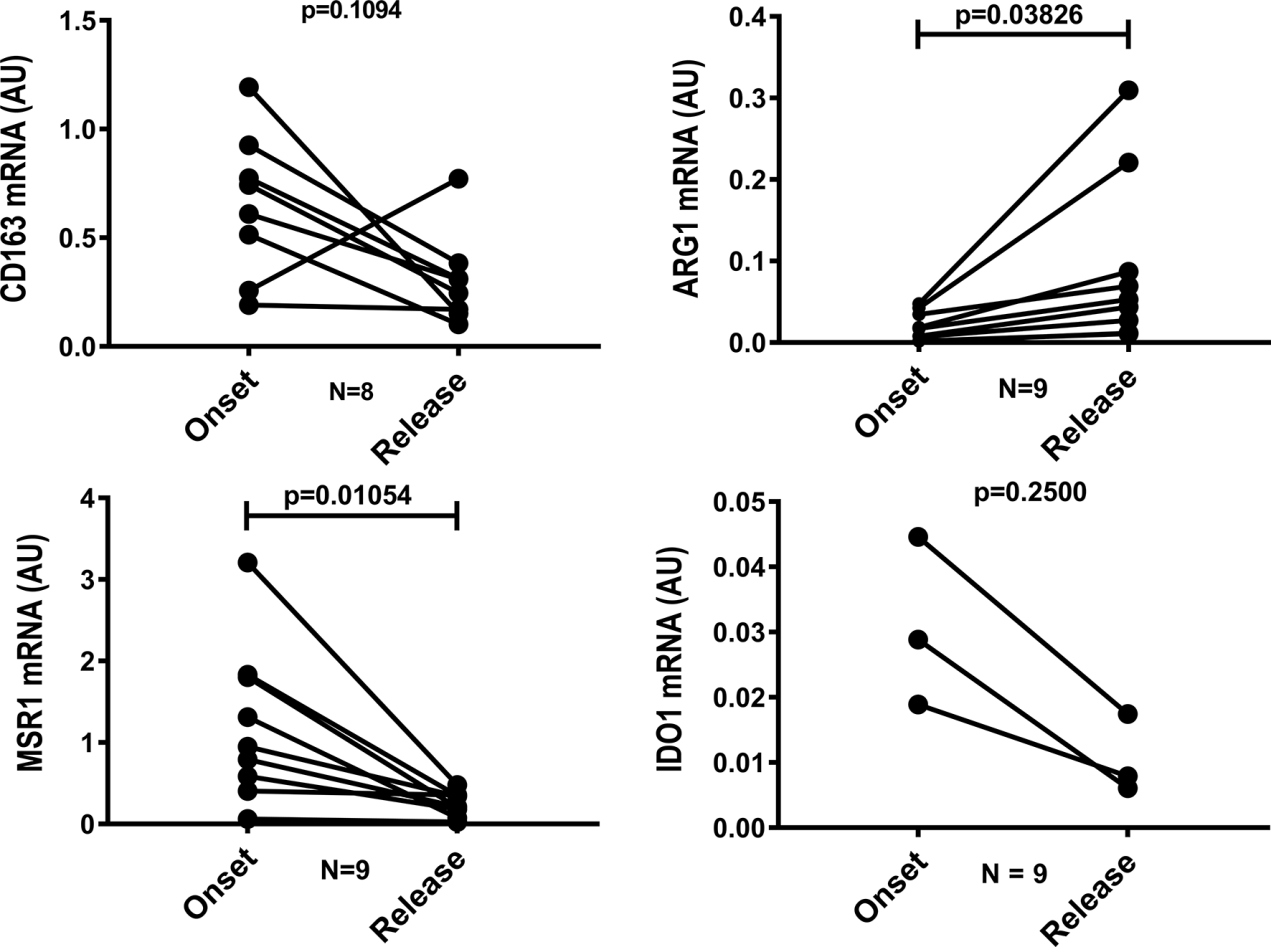
Gene expression of *MSR1* is reduced and *ARG1* increases after 12 doses of MDT. Skin lesions fragments were collected at LL diagnosis (onset) and at release of 12-dose MDT. RNA was extracted, cDNA was synthesized and the expression of *CD163*, *ARG1*, *MSR1* and *IDO1* genes was evaluated by real-time PCR. Graph shows the gene expression of each patient at disease onset and 12-dose MDT release.

To characterize the macrophage phenotype, LL skin lesions were immunostained for CD68, CD163, Arg1 (arginase), MSR1 (SR-A) and IDO-1, which are surface markers predominant in non-treated macrophages (2, 7, 8, 24). We selected representative cases to demonstrate that the inflammatory infiltrates decreases in the release and that anti-inflammatory profile disappears after MDT, with a reduction in CD68+, CD163+, SR-A+, Arg1 and IDO+ cells (**Figure 1**). Gene expression analysis revealed a significant decrease in *MRS1* (that encodes SR-A-) and an increase in *ARG1* expressions (**Figure 2**).

### IFN-γ and CXCL10 (IP-10) Were Reduced in LL Skin Lesion Cells After MDT

**Figure 3:**
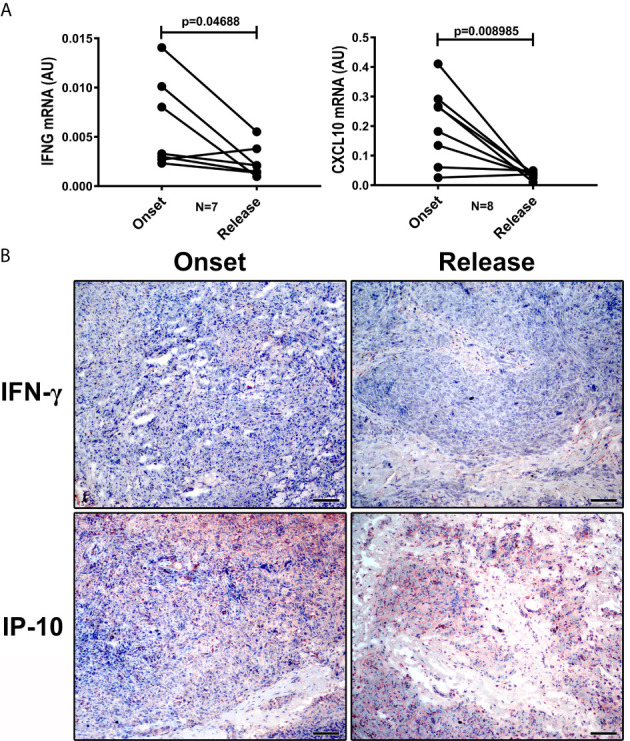
IFN-γ and CXCL10 (IP-10) are reduced in LL skin lesions at 12-dose MDT release. Skin lesions fragments were collected at LL diagnosis (onset) and at release of 12-dose MDT: **(A)** RNA was extracted, cDNA was synthesized and the expression of *IFNG* and *CXCL10* genes was evaluated by real-time PCR. Graphs show the gene expression of each patient at disease onset and 12-dose MDT release. **(B)** Expression of IFN-γ and CXCL10 (IP-10) in the skin lesions was detected by immunohistochemistry. Representative images are shown. Bars: 50 μm.

We evaluated whether 12 doses of MDT increased the presence of pro-inflammatory macrophages in the skin cells of MB patients. We did not observe significant changes in *NOS2A* and *IL15* expression (data not shown). However, IFN-γ and CXCL10 (IP-10) production in skin lesion cells was significantly reduced after MDT, as was observed by gene expression (**Figure 3A**) and histopathology (**Figure 3B**).

### CXCL10 Is Associated With a Reduction in Bacillary Load

**Figure 4:**
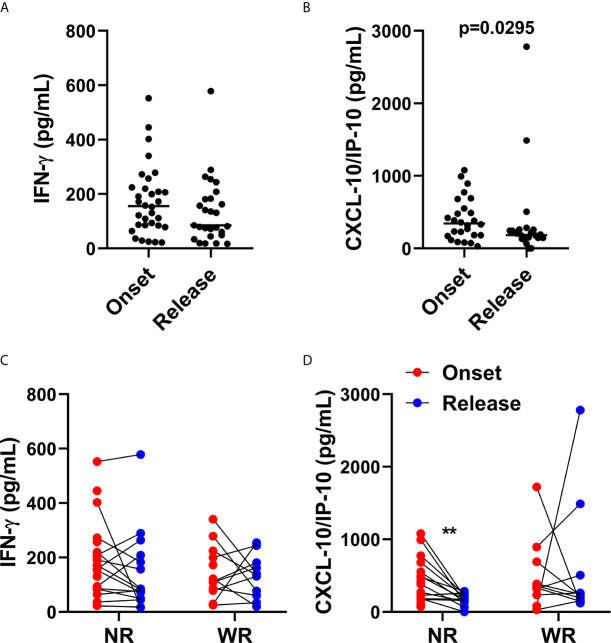
CXCL10 (IP-10) production is associated with reduction of bacilloscopic index at 12-dose MDT release. Anticoagulant-free venous blood samples were collected at LL diagnosis (onset) and at completion of 12 doses of MDT (release), and the sera was aliquoted. Levels of IFN-γ and CXCL10 (IP-10) in the sera were evaluated by ELISA. **(A, B)** Graphs show: IFN-γ **(A)** and CXCL10 (IP-10) **(B)** levels in sera at disease onset and MDT release. **(C, D)** Graphs show: the levels of IFN-γ **(C)** and CXCL10 (IP-10) **(D)** of each patient at disease onset and 12-dose MDT release classified in two groups, one with the patients who presented a reduction in the bacilloscopic index at release (WR, with reduction) and one for the patients who did not reduce the bacilloscopic index after 12-dose MDT (NR, no reduction). **p ≤ 0.01.

We evaluated levels of CXCL10 (IP-10), IFN-γ, IL-6, IL-10, TNF, IL-12p70, TGF-β and IL-17A in LL patient sera. We did not observe significant differences between the levels of IFN-γ (**Figure 4A**), IL-6, IL-10, TNF, IL-12p70, TGF-β and IL-17A (**Supplementary Figure 1**) when comparing between the onset and the release of MDT. CXCL-10 serum levels decreased after MDT (**Figure 4B**).

It is well known that, after MDT, lesions can histologically clear in 2 to 5 years or more (25–27). Moreover, for unexplained reasons, some patients do not reduce their bacillary index, even after 12 doses of MDT. In this context, we divided the recruited volunteers into two groups. The first group was composed of patients who presented a reduction in bacilloscopic index (WR—with reduction) and the other group was composed of patients who did not show a reduction in bacilloscopic index (NR—no reduction) after the release of 12 doses of MDT.

As observed in **Figures 4C, D**, in patients who did not present a reduction in bacilloscopic index, there was a significant decrease in CXCL10 (IP-10) levels in the sera; this was not the case in IFN-γ.

### Reduction in Bacillary Load Is Associated With Increased Production of CXCL10 (IP-10) in Response to *M. leprae*

**Figure 5:**
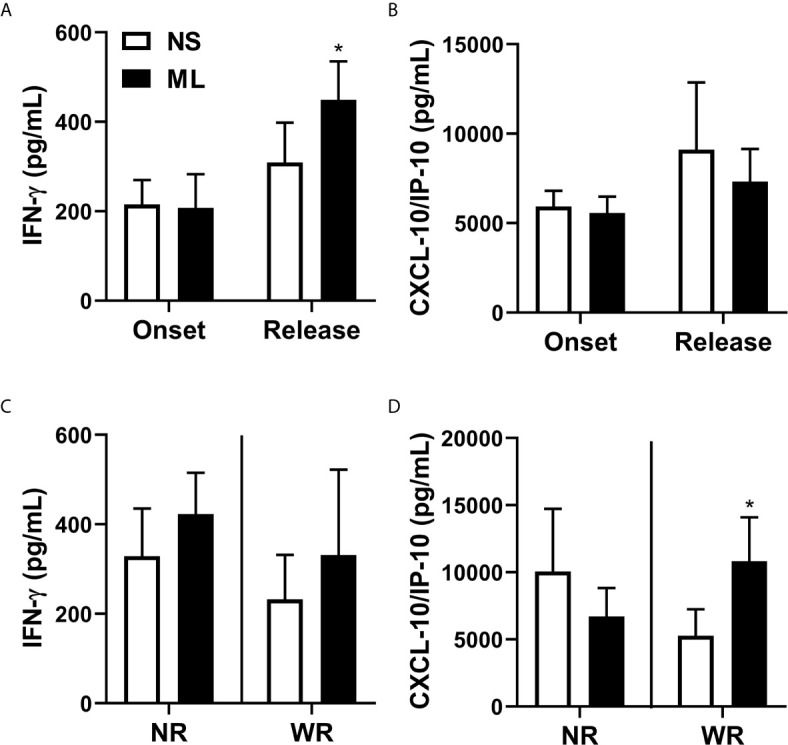
Reduction in bacilloscopic index after 12-dose MDT is associated with increased production of CXCL10 (IP-10). Heparinized venous blood samples were collected at LL diagnosis (onset) and at completion of 12 doses of MDT (release). Whole blood was diluted in AIM-V media and stimulated (ML—stimulated with *M. leprae*) or not (NS—non-stimulated) with 10 μg/mL sonicated *M. leprae* cell antigens for 5 days at 37°C 5% CO_2_. Levels of IFN-γ and CXCL10 (IP-10) in the cell culture supernatants were evaluated by ELISA. **(A, B)** Graphs show: Average ± SEM of IFN-γ **(A)** and CXCL10 (IP-10) **(B)** levels in culture supernatant at disease onset and MDT release. **(C, D)** Graphs show: Average ± SEM of the levels of IFN-γ **(C)** and CXCL10 (IP-10) **(D)** at disease onset and at 12-dose MDT release, classified into two groups. The first group contains patients who presented a reduction in the bacilloscopic index at release (WR, with reduction) and the second group comprises patients who did not reduce the bacilloscopic index after 12-dose MDT (NR, no reduction). *p ≤ 0.05.

In LL, the immune system maneuvers the immune response towards antigen-specific anergy. As observed in **Figure 5A**, levels of IFN-γ were significantly increased after MDT,. CXCL10 (IP-10) levels were not affected by MDT (**Figure 5B**). However, when we separated the NR and WR groups, we observed an increase in CXCL10 (IP-10), but not IFN-γ, in *M. leprae*-stimulated cells compared to non-stimulated cells. This was true for the group that showed a reduced bacilloscopic index after 12 months of MDT (WR) (**Figures 5C, D**).

## Discussion

Antigen-specific T cell responses (Th1 and Th17) have been observed in paucibacillary (PB) patients and are associated with control over *M. leprae* replication (28–32). In contrast, Th2 and T regulatory cells are associated with MB presentations. Although several studies have demonstrated an association between IFN-γ-specific immune responses and protection, the assessment of T cell responses by only measuring IFN-γ may not reflect the protective potential of the response, meaning that other mediators might be involved in the control of the disease. The World Health Organization (WHO) introduced the MDT standardized regimen in 1982, but a high percentage of patients who completed a fixed duration of MDT left with residual skin lesions. In addition, upon MDT completion, patients are considered cured; however, even after MDT, some patients develop leprosy reactions (33).

Despite advances in understanding the pathogenesis of leprosy and perspectives in terms of developing new therapeutic strategies (34), the identification of biomarkers is pivotal for characterizing the immune response in different clinical forms of leprosy, as well as for determining MDT efficacy. There is strong evidence that the immunological response of infected individuals influences not only their susceptibility to *M. leprae* but also the outcome of leprosy. However, little is known regarding the capacity of MDT to modulate a host’s immune response and control the disease, especially in MB patients who present reduced antigen-specific T cell responses. Antigen-specific antibody responses were readily detected in MB patients at the time of diagnosis but were reduced after MDT (35). In this scenario, we can speculate whether the mechanisms associated with control of the disease in MB patients involve increased frequencies of pro-inflammatory macrophages in skin lesions instead of specific T cell responses.

The pathogenesis of MB leprosy involves higher frequency and highly susceptible anti-inflammatory macrophages in skin lesions. Previous studies have demonstrated that MB macrophages have a high phagocytic activity mediated by IL-10 (6, 7, 36). A qPCR performed to evaluate pro- and anti-inflammatory related gene expression in skin lesions of leprosy patients revealed that pro-inflammatory genes *STAT1*, *TNF*, *IFNG*, *IL15* and *CSF2* are increased in PB cells, whereas anti-inflammatory *MSR1* and *PPARG* genes are increased in MB cells (2). Increased expressions of IDO-1, CD163, Arginase 1 and SRA-1 (MSR1) were observed in MB compared to PB cells (7, 8, 24). Although there was a predominance of an alternatively activated phenotype in MB skin lesions at the onset of the disease, the effect of MDT on the macrophage skin phenotype remains uncertain. Here, we investigated the expression of IDO-1, CD163, Arginase 1 and SRA-1 in skin cells of polar lepromatous patients (LL - MB) at onset and after the release of MDT. As expected, treatment with 12 doses of MDT decreased the inflammatory infiltrate in about 90% of the analyzed cases, which was accompanied by a reduction in the expression of scavenger receptors such as SR-A1 (*MSR1*) and CD163, as well as a decrease in IFN-γ and CXCL10 (IP-10) in skin cells associated with an increase in *ARG1*expression.

It is clear that clinically curing the disease is associated not only with bacilli clearance but with the activation of wound healing or tissue repair functions. It is well known that tissue repair comprises a spectrum of overlapping functions that include phagocytosis, secretion of cytokines and growth factors, as well as matrix remodeling (37). In this context, the reduction in proinflammatory mediators in skin cells, such as IFN-γ and CXCL10 (IP-10), could be associated with tissue repair. This hypothesis may be reinforced by an increase in arginase expression. Previous studies have demonstrated that arginase is important for tissue repair (38, 39). Arginase 1 can be produced by cells other than macrophages (40, 41) and, as human macrophages do not produce arginase, human arginase appears to be derived entirely from non-macrophage cell types (40, 42, 43).

Arginase 1 is expressed across a range of cell types involved in wound healing, including keratinocytes (44), fibroblasts (45) and inflammatory cells (46). Arginase 1-mediated metabolism of arginine is an important source of local ornithine, a proline precursor important for collagen synthesis. Previous reports from Singer and Clark (1999) (47) have demonstrated that the main sources of wound collagen are fibroblasts. Although we did not describe the phenotype of Arginase 1-producing cells in MDT-treated LL patients, our data suggest that 12 doses of MDT may modulate *ARG1* expression in LL skin cells. In humans, iNOS activity appears to be indetectable or lower (48). Since Arginase 1 and iNOS compete for the same substrate, the amino acid L-arginine, we evaluated *NOS2* expression in LL skin cells at onset and at release of MDT. No differences were observed between *NOS2* expressions when comparing treated *versus* untreated skin cells.

Cytokines might be identified as good biomarkers of the impact of MDT on the immune system and the effectiveness of treatment. IFN-γ has been studied as a diagnostic host biomarker for leprosy; it is helpful in the differential diagnosis of leprosy from other confounding dermatoses (17), and IFN-γ production in response to *M. leprae* antigens has been used as a marker of the presence of cellular immune responses against bacilli (49–53). Cassirer-Costa and colleagues (2017) (16) have demonstrated increased IFN-γ, IL-6 and IL-10 serum levels in MDT-treated MB patients, but we did not observe significant differences in the sera from LL patients at onset or MDT release. After treatment, increased IFN-γ levels were observed in supernatants from both non-stimulated and *M. leprae*-stimulated cells. The increased production of IFN-γ might be due to Clofazimine, since previous studies have demonstrated that it induces IFN-γ in the cells of treated patients (54, 55). In addition, we cannot exclude the possibility that the destruction of bacilli by MDT may result in enhanced antigen presentation due to higher exposure to antigens, including new antigens such as epitopes contained within LID-1, which could lead to the stimulation of effector T cells and IFN-γ production, even in MB patients (17).

CXCL10 (IP-10) is secreted under pro-inflammatory conditions in response to IFN-γ by various cell types, including leukocytes, monocytes, activated neutrophils, epithelial cells, endothelial cells, stromal cells and keratinocytes (56, 57). In PB skin lesion cells, keratinocytes are the major producer of CXCL10 (IP-10), but this is not the case in MB cells; this is probably due to the absence of high levels of IFN-γ (58). CXCL10 (IP-10) induction may also be mediated by TNF (59). CXCL10 exerts its biological functions *via* CXCR3, which is expressed by activated T cells (60) through the induction of paracrine and/or autocrine signaling; it has been identified as an important prognostic indicator for various diseases (61–64). Here, we observed that patients who did not reduce the bacilloscopic index after MDT showed a decrease in CXCL10 (IP-10) sera levels at release, which was not observed in the sera of patients who presented a reduction in bacillary load after 12 doses of MDT; this suggests that higher levels of CXCL-10 are important for the control of bacillary load. In addition, an analysis of cellular immune responses against *M. leprae* antigens revealed that cells from patients who presented a reduction in the bacilloscopic index after the release of MDT increased the production of CXCL10 (IP-10) in response to *M. leprae*.

We acknowledge that the relatively small sample size due the loss of follow-up of some patients represent a limitation of this study. One limitation of our study is that we could not identify the cells associated with the production of CXCL10 at MDT release. Another limitation is that we only recruited volunteers who did not develop a reaction during treatment. Therefore, our future studies will evaluate the impact of immunological reactions on cell phenotype and function after MDT release.

Hungria and colleagues (2018) (35) claim that the applicability of serology in monitoring treatment efficacy seems limited for MB patients with a high bacillary load at diagnosis, especially for those who are evaluated in a short-term follow-up after the conclusion of their treatment. Therefore, the results presented in this study suggest that CXCL10 (IP-10) may be used to evaluate the efficacy of MDT in MB patients.

## Data Availability Statement

The original contributions presented in the study are included in the article/**Supplementary Material**. Further inquiries can be directed to the corresponding author.

## Ethics Statement

The studies involving human participants were reviewed and approved by Oswaldo Cruz Foundation Ethical Committee. The patients/participants provided their written informed consent to participate in this study.

## Author Contributions

ES, MM, and RP contributed to the design and implementation of the research. HF, MB, and RP contributed to the writing of the manuscript. HF, MM, EO, and MB processed the experimental data and performed the analysis. HF, MM, and RP contributed to the analysis of the results. AS helped with patient care. All authors contributed to the article and approved the submitted version.

## Funding

We thank CAPES, FAPERJ, and CNPq funding institutions for all their financial support. National Council for Scientific and Technological Development (CNPq) - Finance Code 303834/2017-0. Rio de Janeiro Carlos Chagas Filho Research Foundation (FAPERJ) - Finance Code E-26/010.002231/2019.

## Conflict of Interest

The authors declare that the research was conducted in the absence of any commercial or financial relationships that could be construed as a potential conflict of interest.
