# Supplementary figures and images for: Potential Role of CXCL10 in Monitoring Response to Treatment in Leprosy Patients
Source: Front Immunol. 2021 Jul 20;12:662307. doi: 10.3389/fimmu.2021.662307 (PMC8329534; doi:10.3389/fimmu.2021.662307)

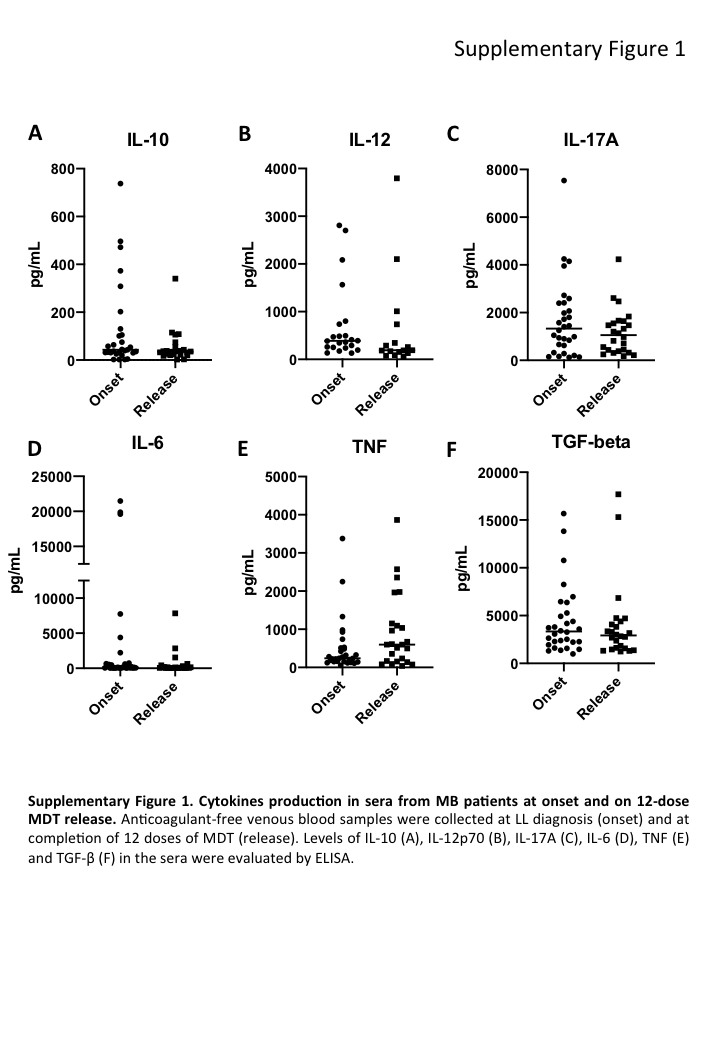

Supplement: Supplementary file 1 [file Image_1.tiff]
